# 4-Guanidinobenzene­sulfonic acid

**DOI:** 10.1107/S1600536809012355

**Published:** 2009-04-08

**Authors:** Wei-Feng Wang, Chang-Mei Wei, Hong-Jun Zhu

**Affiliations:** a College of Science, Nanjing University of Technology, Nanjing 210009, People’s Republic of China; b Department of Chemistry of Huaiyin Teachers College, Jangsu Key Laboratory for the Chemistry of Low-Dimensional Materials, Huaian 223300, People’s Republic of China

## Abstract

In the zwitterionic title compound (systematic name: 4-{[amino(inimio)methyl]amino}benzenesulfonate), C_7_H_9_N_3_O_3_S, the dihedral angle between the plane of the guanidine grouping and the benzene ring system is 44.87 (7)°. The crystal packing is stabilized by inter­molecular N—H⋯O hydrogen bonds involving all the potential donors.

## Related literature

For the synthesis, see: Hofbens & Rath (1981 ▶). For the effect of guanidine salts on protein structure and their inhibitory effect on various physiological activities, see: Miyake *et al.* (2008 ▶).
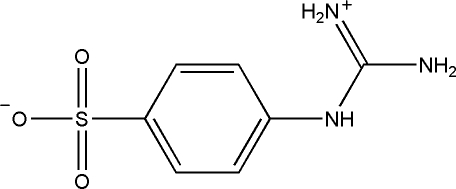


## Experimental

### 

#### Crystal data

C_7_H_9_N_3_O_3_S
*M* *_r_* = 215.24
Orthorhombic, 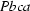

*a* = 7.9967 (9) Å
*b* = 11.9200 (13) Å
*c* = 19.721 (2) Å
*V* = 1879.8 (4) Å^3^
*Z* = 8
Mo *K*α radiation
μ = 0.33 mm^−1^
*T* = 296 K
0.35 × 0.3 × 0.2 mm

#### Data collection

Bruker SMART APEXII CCD diffractometer
Absorption correction: multi-scan (*SADABS*; Bruker, 2000 ▶) *T*_min_ = 0.902, *T*_max_ = 0.944
10292 measured reflections
2156 independent reflections
1544 reflections with *I* > 2σ(*I*)
*R*_int_ = 0.040

#### Refinement

*R*[*F* ^2^ > 2σ(*F* ^2^)] = 0.039
*wR*(*F* ^2^) = 0.110
*S* = 1.02
2156 reflections
163 parameters
H atoms treated by a mixture of independent and constrained refinement
Δρ_max_ = 0.24 e Å^−3^
Δρ_min_ = −0.31 e Å^−3^

### 

Data collection: *APEX2* (Bruker, 2004 ▶); cell refinement: *SAINT* (Bruker, 2004 ▶); data reduction: *SAINT*; program(s) used to solve structure: *SHELXTL* (Sheldrick, 2008 ▶); program(s) used to refine structure: *SHELXTL*; molecular graphics: *SHELXTL*; software used to prepare material for publication: *SHELXTL*.

## Supplementary Material

Crystal structure: contains datablocks global, I. DOI: 10.1107/S1600536809012355/bq2128sup1.cif

Structure factors: contains datablocks I. DOI: 10.1107/S1600536809012355/bq2128Isup2.hkl

Additional supplementary materials:  crystallographic information; 3D view; checkCIF report

## Figures and Tables

**Table 1:** Hydrogen-bond geometry (Å, °)

*D*—H⋯*A*	*D*—H	H⋯*A*	*D*⋯*A*	*D*—H⋯*A*
N1—H5⋯O2^i^	0.80 (3)	2.00 (3)	2.802 (2)	171 (2)
N2—H6⋯O2^ii^	0.88 (3)	2.02 (3)	2.851 (2)	158 (2)
N2—H7⋯O3^iii^	0.89 (3)	2.07 (3)	2.913 (3)	160 (2)
N3—H8⋯O1^iii^	0.91 (3)	2.03 (3)	2.924 (3)	167 (3)
N3—H9⋯O3^iv^	0.83 (3)	2.34 (3)	2.928 (3)	129 (2)

Symmetry codes: (i) 
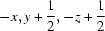
; (ii) 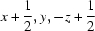; (iii) 
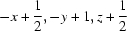
; (iv) 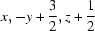.

**Fig. 1.**
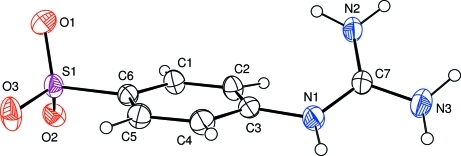
The molecular structure of (I), with atom labels and 40% probability displacement ellipsoids for non-H atoms.

## supplementary crystallographic information

### Comment

Guanidine is known to interact strongly with various substances of biological origin in their molecules forming ionic pairs through ionic bonds and hydrogen bonding. Research on guanidine salts in the field of biochemistry have dealt mainly with their effect on protein structure and inhibitory effect on various physiological activities (Miyake *et al.*,  2008). The full molecule of the title compound, (I), (Fig. 1), is a big hyperconjugation system because the bond lengths of N1—C7 (1.329 (3)Å), C7—N2 (1.313 (3)Å) and C7—N3 (1.327 (3)Å) are averaged, and the bond lengths of S1—O1 (1.4546 (15)Å), S1—O2 (1.4619 (15)Å) and S1—O3 (1.4457 (15)Å) are also averaged. Meanwhile, the bond lengths of C3—N1 (1.327 (3)Å) and C6—S1 (1.770 (2)Å) become shorter than standard values (C—N = 1.47–1.50Å and C—S = 1.82Å). In addition, in (I) C2-C3-N1-C7 form a torsion angle of 42.3 (3)° and C1-C6-S1-O1 form a torsion angle of -69.91 (18)°. The dihedral angle between the plane of the guanidine group and the benzene ring system is 44.87 (7)°, while the dihedral angle between the benzene ring and the adjacent S1O1O2 group is 84.76 (7)°. The crystal packing is stabilized by intermolecular N—H···O hydrogen bonds involving all the potential donors.

### Experimental

The title compound was synthesized by 4-aminobenzenesulfonic acid (3.5 g, 0.02 mol), 50% amino nitrile (3.5 g, 0.04 mol) and 37% hydrochloric acid (3.4 ml) in the ethanol boil point temperature for 24 h with stirring (Hofbens & Rath, 1981). The reaction mixture was reduced pressure distillation to obtain the rough solid, then dissolved in water. The solid residue was filtered and the filtrate was kept at room temperature. Colorless crystals of the title compound were obtained after a few days. The crystal used for data collection was obtained by slow evaporation from a saturated water solution at room temperature.

### Refinement

All of the H atoms were located in a difference synthesis and refined isotropically [aromatic C—H = 0.90 (2)–0.97 (2)Å and N—H = 0.80 (3)–0.91 (3) Å].

### Crystal data

**Table:**

C_7_H_9_N_3_O_3_S	*D*_x_ = 1.521 Mg m^−^^3^
*M**_r_* = 215.24	Melting point > 300 K
Orthorhombic, *P**b**c**a*	Mo *K*α radiation, λ = 0.71073 Å
Hall symbol: -P 2ac 2ab	Cell parameters from 2125 reflections
*a* = 7.9967 (9) Å	θ = 3.2–25.9°
*b* = 11.9200 (13) Å	µ = 0.33 mm^−^^1^
*c* = 19.721 (2) Å	*T* = 296 K
*V* = 1879.8 (4) Å^3^	Block, colourless
*Z* = 8	0.35 × 0.3 × 0.2 mm
*F*(000) = 896	

### Data collection

**Table:**

Bruker SMART APEXII CCD diffractometer	2156 independent reflections
Radiation source: fine-focus sealed tube	1544 reflections with *I* > 2σ(*I*)
graphite	*R*_int_ = 0.040
ω scans	θ_max_ = 27.5°, θ_min_ = 3.2°
Absorption correction: multi-scan (*SADABS*; Bruker, 2000)	*h* = −10→10
*T*_min_ = 0.902, *T*_max_ = 0.944	*k* = −15→13
10292 measured reflections	*l* = −24→25

### Refinement

**Table:**

Refinement on *F*^2^	Primary atom site location: structure-invariant direct methods
Least-squares matrix: full	Secondary atom site location: difference Fourier map
*R*[*F*^2^ > 2σ(*F*^2^)] = 0.039	Hydrogen site location: inferred from neighbouring sites
*wR*(*F*^2^) = 0.110	H atoms treated by a mixture of independent and constrained refinement
*S* = 1.02	*w* = 1/[σ^2^(*F*_o_^2^) + (0.054*P*)^2^ + 0.6089*P*] where *P* = (*F*_o_^2^ + 2*F*_c_^2^)/3
2156 reflections	(Δ/σ)_max_ < 0.001
163 parameters	Δρ_max_ = 0.24 e Å^−^^3^
0 restraints	Δρ_min_ = −0.31 e Å^−^^3^

### Special details

**Table:**

Geometry. All e.s.d.'s (except the e.s.d. in the dihedral angle between two l.s. planes) are estimated using the full covariance matrix. The cell e.s.d.'s are taken into account individually in the estimation of e.s.d.'s in distances, angles and torsion angles; correlations between e.s.d.'s in cell parameters are only used when they are defined by crystal symmetry. An approximate (isotropic) treatment of cell e.s.d.'s is used for estimating e.s.d.'s involving l.s. planes.
Refinement. Refinement of *F*^2^ against ALL reflections. The weighted *R*-factor *wR* and goodness of fit *S* are based on *F*^2^, conventional *R*-factors *R* are based on *F*, with *F* set to zero for negative *F*^2^. The threshold expression of *F*^2^ > σ(*F*^2^) is used only for calculating *R*-factors(gt) *etc*. and is not relevant to the choice of reflections for refinement. *R*-factors based on *F*^2^ are statistically about twice as large as those based on *F*, and *R*- factors based on ALL data will be even larger.

### Fractional atomic coordinates and isotropic or equivalent isotropic displacement parameters (Å^2^)

**Table:**

	*x*	*y*	*z*	*U*_iso_*/*U*_eq_	
C1	0.0397 (3)	0.48689 (18)	0.26624 (10)	0.0329 (5)	
C2	0.0470 (3)	0.53779 (18)	0.32882 (11)	0.0338 (5)	
C3	0.1174 (2)	0.64333 (17)	0.33506 (10)	0.0316 (5)	
C4	0.1778 (3)	0.69826 (18)	0.27833 (11)	0.0356 (5)	
C5	0.1684 (3)	0.64822 (17)	0.21529 (11)	0.0346 (5)	
C6	0.1005 (2)	0.54177 (16)	0.20961 (10)	0.0291 (4)	
C7	0.1541 (3)	0.65879 (18)	0.45879 (10)	0.0349 (5)	
H1	−0.006 (3)	0.4173 (19)	0.2604 (12)	0.044 (6)*	
H2	−0.004 (3)	0.504 (2)	0.3685 (11)	0.043 (6)*	
H3	0.230 (3)	0.765 (2)	0.2812 (11)	0.045 (7)*	
H4	0.210 (3)	0.6900 (18)	0.1779 (13)	0.043 (6)*	
H5	0.101 (3)	0.767 (2)	0.3958 (12)	0.049 (7)*	
H6	0.253 (3)	0.523 (2)	0.4302 (14)	0.055 (8)*	
H7	0.236 (3)	0.529 (2)	0.5062 (14)	0.058 (8)*	
H8	0.178 (3)	0.700 (2)	0.5536 (15)	0.072 (9)*	
H9	0.098 (3)	0.787 (2)	0.5079 (13)	0.052 (8)*	
N1	0.1200 (3)	0.70124 (16)	0.39801 (10)	0.0412 (5)	
N2	0.2066 (3)	0.55499 (17)	0.46566 (11)	0.0439 (5)	
N3	0.1313 (3)	0.7218 (2)	0.51347 (11)	0.0532 (6)	
O1	0.21174 (19)	0.38280 (13)	0.13149 (8)	0.0451 (4)	
O2	−0.07907 (17)	0.43211 (12)	0.12279 (7)	0.0376 (4)	
O3	0.1335 (2)	0.55807 (12)	0.07941 (8)	0.0494 (5)	
S1	0.09167 (7)	0.47441 (4)	0.12971 (2)	0.03204 (18)	

### Atomic displacement parameters (Å^2^)

**Table:**

	*U*^11^	*U*^22^	*U*^33^	*U*^12^	*U*^13^	*U*^23^
C1	0.0413 (11)	0.0274 (11)	0.0300 (11)	−0.0057 (9)	−0.0014 (9)	0.0013 (8)
C2	0.0401 (10)	0.0355 (12)	0.0258 (11)	−0.0029 (9)	0.0007 (9)	0.0027 (9)
C3	0.0386 (10)	0.0276 (10)	0.0287 (11)	0.0059 (9)	−0.0034 (9)	−0.0024 (8)
C4	0.0467 (12)	0.0233 (10)	0.0368 (12)	−0.0024 (9)	−0.0033 (10)	−0.0017 (9)
C5	0.0451 (11)	0.0260 (11)	0.0327 (12)	−0.0013 (10)	0.0034 (10)	0.0034 (9)
C6	0.0348 (10)	0.0256 (10)	0.0268 (10)	0.0012 (8)	−0.0011 (8)	−0.0012 (8)
C7	0.0442 (11)	0.0343 (11)	0.0262 (11)	0.0069 (10)	−0.0028 (9)	−0.0045 (9)
N1	0.0665 (13)	0.0261 (10)	0.0311 (10)	0.0095 (9)	−0.0096 (9)	−0.0053 (8)
N2	0.0691 (13)	0.0396 (11)	0.0231 (10)	0.0176 (10)	−0.0035 (10)	−0.0027 (8)
N3	0.0846 (16)	0.0421 (13)	0.0329 (12)	0.0172 (12)	−0.0051 (11)	−0.0121 (10)
O1	0.0532 (9)	0.0373 (9)	0.0448 (10)	0.0100 (7)	0.0017 (7)	−0.0085 (7)
O2	0.0465 (8)	0.0309 (8)	0.0355 (9)	−0.0020 (7)	−0.0086 (7)	−0.0012 (6)
O3	0.0869 (12)	0.0336 (9)	0.0278 (8)	−0.0105 (9)	0.0088 (8)	0.0034 (6)
S1	0.0474 (3)	0.0247 (3)	0.0240 (3)	−0.0011 (2)	0.0010 (2)	−0.00080 (19)

### Geometric parameters (Å, °)

**Table:**

C1—C2	1.376 (3)	C7—N2	1.313 (3)
C1—C6	1.383 (3)	C7—N3	1.327 (3)
C1—H1	0.91 (2)	C7—N1	1.329 (3)
C2—C3	1.384 (3)	N1—H5	0.80 (3)
C2—H2	0.97 (2)	N2—H6	0.88 (3)
C3—C4	1.383 (3)	N2—H7	0.89 (3)
C3—N1	1.421 (3)	N3—H8	0.91 (3)
C4—C5	1.381 (3)	N3—H9	0.83 (3)
C4—H3	0.90 (2)	O1—S1	1.4546 (15)
C5—C6	1.385 (3)	O2—S1	1.4619 (15)
C5—H4	0.95 (2)	O3—S1	1.4457 (15)
C6—S1	1.770 (2)		
			
C2—C1—C6	120.1 (2)	N2—C7—N3	119.6 (2)
C2—C1—H1	122.0 (15)	N2—C7—N1	121.1 (2)
C6—C1—H1	117.9 (15)	N3—C7—N1	119.3 (2)
C1—C2—C3	119.9 (2)	C7—N1—C3	127.30 (19)
C1—C2—H2	121.6 (14)	C7—N1—H5	117.4 (18)
C3—C2—H2	118.4 (14)	C3—N1—H5	115.2 (18)
C4—C3—C2	120.0 (2)	C7—N2—H6	117.3 (17)
C4—C3—N1	118.13 (19)	C7—N2—H7	120.1 (17)
C2—C3—N1	121.70 (19)	H6—N2—H7	117 (2)
C5—C4—C3	120.3 (2)	C7—N3—H8	119.0 (18)
C5—C4—H3	117.6 (14)	C7—N3—H9	117.9 (19)
C3—C4—H3	122.0 (14)	H8—N3—H9	121 (3)
C4—C5—C6	119.3 (2)	O3—S1—O1	112.45 (10)
C4—C5—H4	116.9 (14)	O3—S1—O2	112.95 (10)
C6—C5—H4	123.7 (14)	O1—S1—O2	111.09 (9)
C1—C6—C5	120.40 (19)	O3—S1—C6	106.78 (9)
C1—C6—S1	119.37 (15)	O1—S1—C6	107.02 (9)
C5—C6—S1	120.22 (15)	O2—S1—C6	106.07 (9)
			
C6—C1—C2—C3	−0.9 (3)	N2—C7—N1—C3	6.7 (4)
C1—C2—C3—C4	1.1 (3)	N3—C7—N1—C3	−171.9 (2)
C1—C2—C3—N1	176.8 (2)	C4—C3—N1—C7	−141.9 (2)
C2—C3—C4—C5	−0.1 (3)	C2—C3—N1—C7	42.3 (3)
N1—C3—C4—C5	−176.0 (2)	C1—C6—S1—O3	169.46 (17)
C3—C4—C5—C6	−1.1 (3)	C5—C6—S1—O3	−10.8 (2)
C2—C1—C6—C5	−0.2 (3)	C1—C6—S1—O1	−69.91 (18)
C2—C1—C6—S1	179.46 (16)	C5—C6—S1—O1	109.79 (18)
C4—C5—C6—C1	1.2 (3)	C1—C6—S1—O2	48.76 (18)
C4—C5—C6—S1	−178.46 (16)	C5—C6—S1—O2	−131.54 (17)

### Hydrogen-bond geometry (Å, °)

**Table:**

*D*—H···*A*	*D*—H	H···*A*	*D*···*A*	*D*—H···*A*
N1—H5···O2^i^	0.80 (3)	2.00 (3)	2.802 (2)	171 (2)
N2—H6···O2^ii^	0.88 (3)	2.02 (3)	2.851 (2)	158 (2)
N2—H7···O3^iii^	0.89 (3)	2.07 (3)	2.913 (3)	160 (2)
N3—H8···O1^iii^	0.91 (3)	2.03 (3)	2.924 (3)	167 (3)
N3—H9···O3^iv^	0.83 (3)	2.34 (3)	2.928 (3)	129 (2)

Symmetry codes: (i) −*x*, *y*+1/2, −*z*+1/2; (ii) *x*+1/2, *y*, −*z*+1/2; (iii) −*x*+1/2, −*y*+1, *z*+1/2; (iv) *x*, −*y*+3/2, *z*+1/2.
